# Community Impacts of *Prosopis juliflora* Invasion: Biogeographic and Congeneric Comparisons

**DOI:** 10.1371/journal.pone.0044966

**Published:** 2012-09-12

**Authors:** Rajwant Kaur, Wilfredo L. Gonzáles, Luis Daniel Llambi, Pascual J. Soriano, Ragan M. Callaway, Marnie E. Rout, Timothy J. Gallaher, Inderjit

**Affiliations:** 1 Department of Environmental Studies, Centre for Environmental Management of Degraded Ecosystems (CEMDE), University of Delhi, Delhi, India; 2 Laboratorio de Ecología Evolutiva, LID, Facultad de Ciencias y Filosofía, Universidad Peruana Cayetano Heredia, Lima, Perú; 3 Instituto de Ciencias Ambientales y Ecológicas, Universidad de Los Andes, La Hechicera, Mérida, Venezuela; 4 Division of Biological Sciences, University of Montana, Missoula, Montana, United States of America; 5 Botany Department, University of Hawaii at Manoa, Honolulu, Hawaii, United States of America; Institute of Botany, Czech Academy of Sciences, Czech Republic

## Abstract

We coordinated biogeographical comparisons of the impacts of an exotic invasive tree in its native and non-native ranges with a congeneric comparison in the non-native range. *Prosopis juliflora* is taxonomically complicated and with *P. pallida* forms the *P. juliflora* complex. Thus we sampled *P. juliflora* in its native Venezuela, and also located two field sites in Peru, the native range of *Prosopis pallida.* Canopies of *Prosopis juliflora*, a native of the New World but an invader in many other regions, had facilitative effects on the diversity of other species in its native Venezuela, and *P. pallida* had both negative and positive effects depending on the year, (overall neutral effects) in its native Peru. However, in India and Hawaii, USA, where *P. juliflora* is an aggressive invader, canopy effects were consistently and strongly negative on species richness. *Prosopis cineraria*, a native to India, had much weaker effects on species richness in India than *P. juliflora*. We carried out multiple congeneric comparisons between *P. juliflora* and *P. cineraria*, and found that soil from the rhizosphere of *P. juliflora* had higher extractable phosphorus, soluble salts and total phenolics than *P. cineraria* rhizosphere soils. Experimentally applied *P. juliflora* litter caused far greater mortality of native Indian species than litter from *P. cineraria*. *Prosopis juliflora* leaf leachate had neutral to negative effects on root growth of three common crop species of north-west India whereas *P. cineraria* leaf leachate had positive effects. *Prosopis juliflora* leaf leachate also had higher concentrations of total phenolics and L-tryptophan than *P. cineraria,* suggesting a potential allelopathic mechanism for the congeneric differences. Our results also suggest the possibility of regional evolutionary trajectories among competitors and that recent mixing of species from different trajectories has the potential to disrupt evolved interactions among native species.

## Introduction

**Figure 1:**
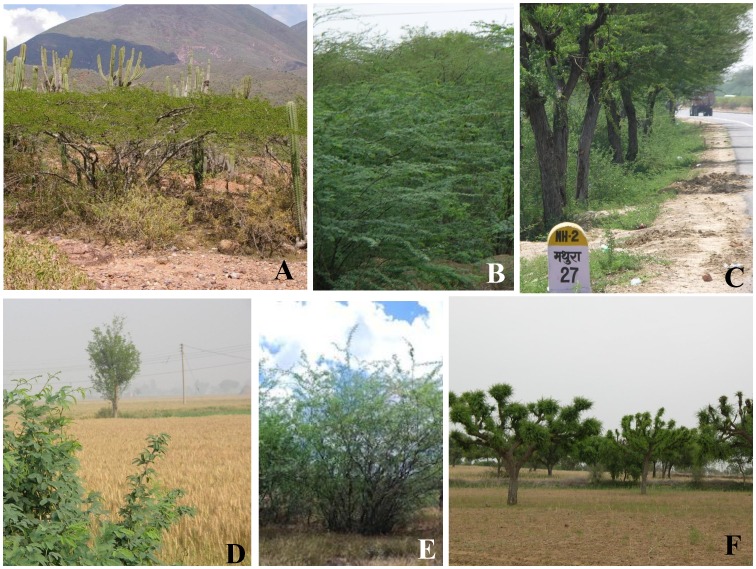
*Prosopis juliflora* in its native range of Venezuela (A); the invaded range of Haryana, India (B), along the National Highway to Rajasthan (C), at the boundaries of an agricultural field in India (D), and in Hawaii, USA (E); *Prosopis cineraria* in its native range, Rajasthan, India (F). Photo credits: Pascual J. Soriano (A); Inderjit (B, C, D and F) and Timothy J. Gallaher (E).

Why some exotic plants, when introduced to a new part of the world, become far more abundant and have greater impact than in their native range is one of the most puzzling questions in ecology [1], [2]. *Prosopis juliflora* (Swartz) DC appears to be one of these species. Several species of *Prosopis* have been introduced to different parts of the world and four – *P. glandulosa, P. velutina, P. juliflora* and *P. pallida* – have become invasive [3]. *Prosopis juliflora* and *P. pallida* are tropical species and have become serious invasives in many parts of Africa, Middle East and Indian subcontinent. *Prosopis juliflora* is native to Central America, northern South America and the Caribbean islands [4] (Figure 1A), and *P. pallida* is native to northern South America. In their native ranges both species appear to coexist with large numbers of other native species. A significant amount of confusion exists in the identification of *P*. *juliflora* and *P*. *pallida* [3]. For example, a species previously identified as *P. juliflora* in Peruvian-Ecuadorian coast (one of the native ranges) is now identified as *P. pallida* or *P. limensis* [5]. We used morphological characters to confirm identity of *P. juliflora* [3] and treated both *P. juliflora* and *P. pallida* as a complex, “*P. juliflora* s. lat. (incl. *P. pallida*)” and recognize this as the *P. juliflora* complex.

*P. juliflora* is a major invasive species in India, and has also invaded other regions throughout the world including Saharan and southern Africa, the Middle East, Pakistan, India, and Hawaii (USA) [3] where it appears to strongly suppress species native to those regions. *Prosopis juliflora* forms pure stands in its invaded range in India, and occurs in forests, wastelands and at the boundaries of crop fields (Figure 1B, C, D). *Prosopis juliflora* also occurs in saline habitats in Hawaii USA (Figure 1E).

In its native range, densities of *P. juliflora* can be high relative to other leguminous shrubs and trees, but its canopies can have much stronger facilitative effects on neighbors than other leguminous tree species [6]. Many other *Prosopis* species, in their native ranges, create “resource islands” with higher concentrations of organic matter, nitrogen, phosphorus and potassium beneath their canopies and behave as strong facilitators of other species [7]–[17]. Accordingly, *Prosopis cineraria*, indigenous to North-Western India, can facilitate native species [18]. Farmers keep *P. cineraria* in their fields because their crops grow better under the trees than in the open fields [19] (Figure 1F). Aggarwal et al. [19] also found that soil nitrogen, phosphorus, and potassium were higher under *P. cineraria* canopies than in open fields, and that the biomass of *Pennesetum typhoides* was three times higher when grown in *P. cineraria* soil than in open soil. Adding nutrients to non-*P. cineraria* soil reduced these differences, but *P. typhoides* grown in *P. cineraria* soils was always at least two times larger than when grown in soil from outside of *P. cineraria* canopies regardless of nutrient additions. These studies suggest that in their native ranges, *Prosopis* species often have neutral to positive effects on the species beneath them.

*Prosopis juliflora* was first introduced to India in 1877 where it has become invasive. However it is also a source of fuel wood, fodder, charcoal and timber [3]. In India, Aggarwal et al. [18] found that the canopies of the invasive *P. juliflora* had far fewer understory species than any of four other species measured, whereas the native congener, *P. cineraria*, was associated with higher subcanopy diversity than any other species. In the Arabian Peninsula where *P. juliflora* is invasive has strong negative impacts on native species despite increases in the concentrations of some nutrients in subcanopy soil [20].

Exotic plants can also have different impacts on nutrient cycling than native species through nutrient release and biochemical effects [21]–[24]; suggesting the potential importance of comparisons of the effects of *P. juliflora* to its native congener, *P. cineraria*, on the chemical characteristics of soil. Inderjit et al. [25] compared the effects of soils from the rhizospheres of *P. juliflora* and *P. cineraria* and found that soil under *P. juliflora* had higher concentrations of total phenolics and inhibited total biomass of *Bambusa arundinacea* more than soil from under the native congener. Goel et al. [26] reported allelopathic potential of *P. juliflora* leaf leachate prepared in hot water and decomposing litter residues. The amino acid L-tryptophan has been isolated from foliage leachate of *P. juliflora* and has been shown to have allelopathic potential on *Echinochloa crus-galli* in filter paper bioassays [27].

The apparent contrasts in the ecology of *P. juliflora* in its native and non-native ranges, and between the effects of *P. juliflora* and *P. cineraria* in the latter’s native range, is curious. This biogeographical contrast parallels those exhibited by many other invasive species and raises questions about interactions among species from different parts of the world might be affected by evolutionary mismatches [1], [28]. We hypothesized that *P. juliflora* in its invaded ranges has (i) greater negative impacts on species richness compared to its native range and compared to a congeneric species in its invaded range, (ii) more pronounced effects on soil chemical characteristics and allelochemical pools than a congener, and (iii) greater inhibition of species native to the invaded ranges in soil and leaf leachate experiments than a congener. We tackled this general issue by comparing (1) subcanopy species richness under *P. pallida* canopies with species richness in open areas, in its native range Peru and *P. juliflora* in its native range Venezuela, and two parts of its non-native range, India and Hawaii, (2) subcanopy species richness under *P. juliflora* and the Indian native *P. cineraria* in India, (3) the chemical characteristics (inorganic ions and total phenolics) of the soil from the rhizosphere of the two congeners, (4) the effects of litter from the two congeners on a suite of native Indian species, and (5) the effect of soil amended with leaf leachate of the two congeners on local crop species in India.

## Materials and Methods

### *Prosopis juliflora* and Species Richness in Native and Non-native Ranges

*Prosopis juliflora* is part of the *Prosopis juliflora*-*pallida* complex, a taxonomically complicated, interrelated, and controversial group both in the native and non-native ranges of species in this complex [5]. Because of this pervasive taxonomic confusion, we took a conservative approach to sampling in Venezuela, native range of *P. juliflora*, and in Peru, the native range of what is most likely to be *Prosopis pallida* [5]. Our intent was to make a broader measurement of the impacts of the *Prosopis juliflora* complex in the native ranges by using both ends of the species that make up the *P. juliflora-pallida* complex. We located two field sites in Peru, (i) Chulucanas (S 05°11′ 49.4″; W 080°16′ 53.3″; 174 m) and (ii) Catacaos (S 05°27′ 06.5″; W 80°14′ 37.2″; 253 m). We located two field sites in Venezuela, in the native range of *Prosopis juliflora* [5], (i) Mucumi (N 08°30′ 10.0″; W 71°21′ 53.0″; 1000 m) and (ii) Puente Real (N 08°28′ 41.0″; W 71°24′ 38.0″; 650 m). Both sites are within the Lagunillas Semi-arid Enclave, which is an inter-Andean enclave, the largest in Venezuela with an area of 262 km^2^. The vegetation at both study sites is classified as thorn scrub. In these sites the dominant elements in the upper stratum were the thorny shrubs *Prosopis juliflora* and *Acacia macracantha*, which form an open and discontinuous canopy up to 3 m in height. The columnar cacti *Stenocereus griseus*, *Pilosocereus tillianus* and *Cereus repandus* are emergent elements (up to 5–7 m in height), which tend to be spatially associated with these leguminous shrubs. The intermediate stratum (up to 50 cm) is formed by shrubs of *Croton* spp., *Capparis odoratissima*, herb *Hibiscus phoeniceus*, cacti *Opuntia caribea*, *Opuntia depauperata*, *Opuntia* aff. *elatior* and *Solanum* spp. A bromeliacea of the *Pitcairnia* genus, the herbs *Jatropha gossypifolia*, *Cnidosculus urens* and *Evolvulus* sp., and annual plant species were also present. Three field sites were located in India and two sites were located in Honolulu, Hawaii, non-native ranges of *P. juliflora*. The sites located in India were: (i) Delhi (N 28°35′ 58.6″; E 077°10′ 15.6″; 233 m), (ii) Kutch, Gujarat (N 23°55′ 58.2″; E 069°48′ 50.4″; 407 m), and (iii) Jaipur, Rajasthan (N 27°01′ 00.3″; E 075°46′ 12.9″; 484 m). The three Indian sites were chosen to represent a range of climates, with Delhi, Gujarat and Rajasthan having humid subtropical, tropical monsoon and tropical arid climates, respectively. The two sites in Hawaii were: (i) Sand Island (N 21° 18′ 12.5″ W 157° 52′ 51.7″ and (ii) Iroquois Point-Hau Bush (N 21° 19′ 24.9″; W 157° 58′ 16.7″ and N 21° 18′ 20.9″; W 157° 1′ 52.5″), both at sea level.

At each site in Peru, we randomly placed 1 m^2^ quadrats in areas of large patches of *P. pallida* and where *P. pallida* was not present, what we refer to as ‘open areas’. We recorded the number of species in each plot. In 2008, we sampled 96 pairs of plots at Chulucanas and 92 pairs at Catacaos. In 2009, we sampled 72 pairs in Chulucanas and 120 pairs at Catacaos. At each site in Venezuela in July 2012, we randomly placed one 1 m^2^ quadrat under each of 15 different *P. juliflora* trees and in open grassland adjacent to each tree where *P. juliflora* was not present and recorded the number of species in each plot. At the Delhi site *P. juliflora* primarily occurs in dense stands (3.6 trees per 25 m^2^). Here in May 2010 we established 10 randomly placed 5×5 m^2^ quadrats under *P. juliflora*, with each plot representing the understory of a different set of several trees, and 10 5×5 m^2^ quadrats in open area outside of the *P. juliflora* stands. At Gujarat, the site was a scrub forest of *P. juliflora* (1.4 trees per 25 m^2^) in which 7 randomly placed 5×5 m^2^ plots were located under *P. juliflora* and 7 in open areas away from *P. juliflora* canopy, during June 2008. At Rajasthan, *P. juliflora* tended to be smaller and occur in very large closed canopy patches precluding pairing local sub canopies and open patches (4.4 trees per 25 m^2^). Thus 10 randomly placed 5×5 m^2^ quadrats were located under *P. juliflora* roughly in the centre of a typical stand that was several kilometres in diameter, with control quadrats (no *P. juliflora*) approximately 2 km away, from the quadrats with *P. juliflora* but in what appeared to be very similar environmental conditions. Sampling was in June 2010. At Delhi and Rajasthan, open areas were selected outside *P. juliflora* stands because these stands were closed-canopy woodlands rather than savanna-like vegetation. At Delhi, the open area was characterized by few far-separated trees of *Albizzia lebbeck, Balanites roxburghii* with interspersed patches of shrub species including *Grewia* sp., *Capparis sepiaria*, *Capparis decidua* and *Zizyphus nummularia*, and grasses including *Cenchrus ciliaris, Chrysopogon fulvus* and *Heteropogon* sp. At Rajasthan also, open area consisted of far-separated tree species (*Dalbergia sissoo* and *Azadirachta indica* ) with interspersed patches of shrubs (*Zizyphus* sp., *Lantana camara*, *Morus* sp.) and grasses (*Saccharum* sp. and *Cenchrus ciliaris*). However at Gujarat, open areas and *P. juliflora* subcanopy quadrats were interspersed within the *P. juliflora* stand due to non-overlapping canopies and open areas included tree species (*Acacia senegal and Wrightia tinctoria*), shrub species (*Euphorbia caducifolia*, *Commiphora wightii* and *Grewia* sp.) and herb species (*Tephrosia* sp., *Rhynchosia* sp. and *Cenchrus* sp.).

*Prosopis juliflora* was first recorded at the Sand Island site in Hawaii in the early 1970’s and today this area holds the largest population of *Prosopis juliflora* and *P. juliflora x P. pallida* hybrids in Hawaii. The second site, Iroquois Point-Hau Bush, is within a small stretch of undeveloped coastal area. Its location is approximately 10 km to the west of the Sand Island site with a very similar climate to that of Sand Island. Within this area, there is a large established population of *P. pallida* with *P. juliflora* and hybrids found near the coast. In October-November 2011, 5×5 m^2^ quadrats were placed at each site in areas of dense infestation of *P. juliflora* and areas where it was not present. The number of plant species present in each quadrat was counted.

Our initial plan was to use 1 m^2^ plots in the two non-native ranges, India and Hawaii, as we did in the native range, of *P. juliflora*, Venezuela and of *P. pallida*, Peru. However, the number of species present was much lower under canopies of *P. juliflora* in the non-native ranges, India and Hawaii. We therefore used larger plots to avoid large numbers of zeros in these plots. We did intra-site species richness comparisons under *P. juliflora* canopies and nearby open areas where we used identical sampling methods, at each site in native and invaded range and then compared the effect of *P. juliflora* on species richness observed in its invaded ranges to those observed in its native range.

Species richness in the open and under *P. juliflora* canopies at each site was compared using independent samples t-tests, one-way ANOVA or Mann-Whitney U tests [29].

No specific permits were required for the field studies carried out in Venezuela, India or Hawaii, USA; and field studies in these areas did not involve endangered or protected species. For the field studies carried out in Peru, permission was obtained from the Agriculture Ministry – Perú (N°145-2008-INRENA-IFFS-DCB). The field study did not involve endangered or protected species.

### Effects of *Prosopis* Congeners on Species Richness

We compared species richness beneath *P. juliflora* canopies to that beneath canopies of the native congener, *P. cineraria*, at Deer Park at Patiala, Punjab, India (N 30°17′ 00.2″; E 076°23′ 07.5″; 239 m). The area has a sub-tropical climate and total annual rainfall during the last 5 years of 703.6 mm. The selected site has been invaded by *P. juliflora* that reach heights of 12 to 14 m and densities of 1.1 trees per 25 m^2^. *Prosopis cineraria* occurs there as scattered individuals reaching heights of 4 to 9 m and densities of less than 0.4 trees per 25 m^2^. The canopy areas occupied by individual *P. juliflora* and *P. cineraria* trees were 139.9 and 20.8 m^2^, respectively. We used slightly smaller quadrats (4×4 m^2^) for measurements at this site so that a single quadrat represented a single tree either of *P. juliflora* or *P. cineraria*. In October 2010, we randomly selected 10 trees of *P. juliflora* or *P. cineraria* of similar sizes and laid a 16 m^2^ quadrat under the canopy of each tree keeping the trunk in the centre, and recorded the number of plant species in each quadrat. We also located 10 randomly located 16 m^2^ quadrats in open areas where neither *P. juliflora* nor *P. cineraria* was present. Differences in species richness in open, under *P. juliflora* and *P. cineraria* were tested using one-way ANOVA and post-hoc Tukey’s test [30].

### Effects of Congeners on Soil Chemistry

We also compared soils from the rhizospheres of *P. juliflora* and *P. cineraria* at Deer Park. We did sampling at this site during two time periods, November 2009 and March 2011. During November 2009, soil was collected from rhizosphere of 4 individual trees of each *Prosopis* species and from 4 nearby open locations (n = 4 for each of the three treatments). Soil was collected near tree trunk to a depth of 30 cm and presence of a mesh of horizontal and vertical roots in the pit confirmed that it was rhizosphere soil. In March 2011, soil was collected from rhizosphere of 6 individual trees of each *Prosopis* species and from 6 nearby open locations (n = 6 for each of the three treatments). Soil was collected from 3 points around the tree trunk of each tree selected (no. of subsamples per tree = 3). The soil was then air-dried, sieved (2 mm), and stored in paper bags for later analyses. Five g soil from each sample was shaken with 25 mL water for 1 h followed by filtration through Whatman # 1 filter papers. pH and electrical conductivity were measured in soil extracts using a digital meter (Metrex Sci. Instr. Pvt. Ltd., India). Organic carbon was estimated following methods of Walkley and Black [31]. Total organic nitrogen was estimated using semi-micro Kjeldahl digestion [32]. We estimated PO_4_^3–^P in 2.5% acetic acid soil extracts through molybdenum blue method [32]. Total phenolic content of soils was measured with the Folin-Ciocalteu method [33].

Data from collection during two time periods were combined (n = 10 for each treatment) and, tested for the differences among open, *P. juliflora* and *P. cineraria* soils for each soil chemical property using one-way ANOVA and post-hoc Tukey’s tests [29].

### Effects of Leaf Litter from *Prosopis* Congeners on Other Species

We first tested the effects of leaf litter at The University of Montana in a greenhouse experiment. We compared the effects of litter of *P. juliflora* and *P. cineraria* on the mortality of six native Indian species (*Acacia nilotica, Brassica campestris, Brassica juncea, Chloris dolichostachya, Dalbergia sissoo* and *Prosopis cineraria*). Litter from the *Prosopis* congeners were collected from three different trees in Aravalli Biodiversity Park, Delhi, India (N 28° 33′ 18.9″; E 077° 08′ 56.8″; 247 m), mixed, and then air dried. Rocket pots (200 cm^3^, 3 cm diam) were filled with 20/30 grit silica sand, and 5–10 seeds were planted 5 mm below the surface of the sand in each pot. We put 1.0 gm of litter from either *P. juliflora* or *P. cineraria* on the surface of the substrate, so that the seeds would have to germinate and grow through the leaves as they would through litter in the field. As seeds germinated they were thinned continually so as to keep only the largest seedling in each pot. The experiment started on 8 January 2010 and ended on 9 February 2010. We summed the total final mortality for all of the remaining largest seedlings in all pots for each species, and then compared treatments using the means of mortality for the six species as replicates in an ANOVA testing treatments (control, *P. juliflora*, *P. cineraria*) and followed by Tukey HSD tests [29].

### Effects of Leaf Leachate from *Prosopis* Congeners on Other Species

We also tested the effects of soil amended with leachate from leaves of the two congeners on three crop species, *B. campestris*, *B. juncea* and *Sorghum bicolor*, at the University of Delhi. We used these three crop species because these are common crop species in north-west India where *P. juliflora* has invaded agricultural fields or is present at the boundaries. *Prosopis cineraria* is often present in crop fields (Figure 1F). Leaves of the two *Prosopis* species were collected from the same sites as in the previous experiment and air dried prior to use. Ten g of air dried leaves of each were soaked separately in 100 mL distilled water for 14 h in the dark followed by filtration through Whatman # 1 filter paper. Soil (sandy loam) for the bioassay was collected from an area not occupied by *P. juliflora*, and at the same site where leaves were collected. 50g air-dried soil was weighed into a 9 cm Petri dish and treated with 15 mL distilled water, 5x, 2x, or 1x diluted *P. juliflora* or *P. cineraria* leaf leachate which yielded concentrations of leaf leachate in soil at 0, 60, 150 or 300 µL of leaf leachate/g soil. *Brassica campestris* and *B. juncea* seeds were washed with distilled water before use. *Sorghum bicolor* seeds were surface-sterilized by soaking in 1% sodium hypochlorite for 10 min and then rinsing in distilled water 20 times before use. For each of the seven treatments and three test species, we used six Petri dishes. Ten seeds of *B. campestris*, *B. juncea*, or *S. bicolor* were placed in each Petri dish and kept at 20–23°C. Seven days after treatment application we measured the shoot and root lengths of the germinants. Two-way ANOVA was used to test the effect of leachate source and concentration as fixed factors on root length. We first compared the effect of *P. juliflora* leachate or *P. cineraria* leachate treatment (each concentration) with control and then the effect of *P. cineraria* leachate with *P. juliflora* leachate applied at identical concentrations, using independent samples t-tests [30].We also correlated leachate concentration with root length for each bioassay species [30].

### Effects of Microbes in Litter of Congeners on Other Species

The biochemical effects of litter from different species or different regions could be confounded by different effects of the pathogens in the litter. Therefore we cultivated bacteria and fungi from the leaves of the two *Prosopis* congeners and applied them to two species native to India, *Brassica juncea* and *Chloris dolichostachya*. Cultivation of bacterial and fungal communities of *Prosopis* leaves was achieved by streaking the leaves of each congener (in triplicate) across the surface of several standard microbiological media agar plates. Plates were incubated at 37°C for 1 week in order to observe any potential bacterial or fungal growth. Five different media were used to cultivate microbial growth. The media used to select for bacteria were R2 agar [34], Minimal Agar, and Nutrient Agar [35] treated with 300 mg L^−1^of chloramphenicol to inhibit fungal growth. The media used to select for fungi were Mycobiotic Agar and Potato Dextrose Agar [35]. After the incubation period, individual colonies were selected from the media plates and were used to make separate bacterial and fungal community inocula from each *Prosopis* congener. Each inoculum (either bacterial or fungal) was prepared by aseptically transferring individual microbial colonies into sterile Eppendorf tubes (2 mL volume) filled with 1.5 mL sterile Milli-Q water, and gently vortexed. Mixing 0.5 mL of the bacterial community inoculum with 0.5 mL of the fungal community inoculum made a third composite microbial inoculum. Individual seedlings of *B. juncea* and *C. dolichostachya* were germinated in 200 cm^3^ rocket pots filled with 20/30 grit silica sand, and after two weeks were inoculated with the bacterial, fungal, or composite microbial inocula, with n = 15 for each species and treatment combination. We measured survival of these seedlings 20 days after inoculation.

### Chemical Characteristics of Soil Amended with Leaf Leachate of Two Congeners

To determine the effect of leaf leachate treatment on soil chemistry, 50g soil in a 9 cm Petri dish was amended with 15 mL distilled water (control) and 1x diluted *P. juliflora* or *P. cineraria* leaf leachate (0 and 300 µL *P. juliflora* or *P. cineraria* leaf leachate/g soil) as described above. Six replicate Petri dishes were used per treatment. Immediately after treatment, lids of Petri dishes were removed and soil was allowed to air-dry for 48 h. Soil was then analysed for pH, electrical conductivity, organic carbon, PO_4_^3–^P, and total organic N as described above. We tested the difference among *P. juliflora, P. cineraria* leaf leachate amended and unamended soil for each soil variable using one-way ANOVA and post-hoc Tukey’s test [30].

### Effects of Leaf Leachate and Leaf Litter of *Prosopis* Congeners on Soil Phenolic Content

Soil amended with leaf leachates of *P. juliflora* or *P. cineraria*, as described above, was also analyzed for total phenolic concentration in addition to other chemical properties. In a separate experiment, we added 60 mg of air dried leaves of *P. juliflora* or *P. cineraria* to 5 g soil and moistened the soil with 2 ml distilled water. Litter-amended soil was incubated at 30–34°C for 0, 1, 2, 3, 4, 6, 8, 10 or 14 d. Each treatment and the control (unamended soil) were replicated four times. Measurement of total phenolics in soils from two experiments was performed as described above. We tested the differences among *P. juliflora* litter, *P. cineraria* litter, leaf leachate amendments, and untreated soil for total phenolics using one-way ANOVA and post-hoc Tukey’s test [30].

### L-tryptophan Recovery from Leaf Leachate Amended Soil

Nakano et al. [27] reported that the allelopathic effects of *P. juliflora* foliage appeared to be caused by L-tryptophan, thus we suspected it might play a role in the effects we measured for litter and leaf leachates. For this, we quantified L-tryptophan in leaf leachates from *P. juliflora* or *P. cineraria* and in soil amended with these leachates. Two ml of leaf leachate from *P. juliflora* or *P. cineraria* was added to 10 mL methanol, and L-tryptophan was quantified with high-performance liquid chromatography (HPLC). Five g soil was treated with 2 mL leaf leachate from *P. juliflora, P. cineraria* or with distilled water and then immediately extracted in 10 mL methanol by shaking for 20 min followed by centrifugation at 4500 rpm for 10 min. The supernatant was filtered through 0.2 µm PES syringe filters directly into HPLC vials.

L-tryptophan level in methanol extract of each soil and leachate sample was determined by using HPLC (Waters Corp., Milford, U.S.A), employing a Waters Spherisorb 5 µm ODS2 column (4.6×250 mm Analytical Column). The mobile phase consisted of 50 mM sodiumphosphate buffer, pH 3.5 containing 20% methanol (v/v) and flow rate was 1 mL/min. L-tryptophan was detected at 214 nm using a photodiode array detector. 10–20 µL of sample was injected in the partial loop needle overfill mode, run time for each sample was 20 min, and the retention time of L-tryptophan was 8.17 min with 0.27% RSD. L-tryptophan concentration in leaf leachates from two congeners was compared using independent samples t-test [30].

## Results

### *Prosopis juliflora* and Species Richness in Native and Non-native Ranges

**Figure 2:**
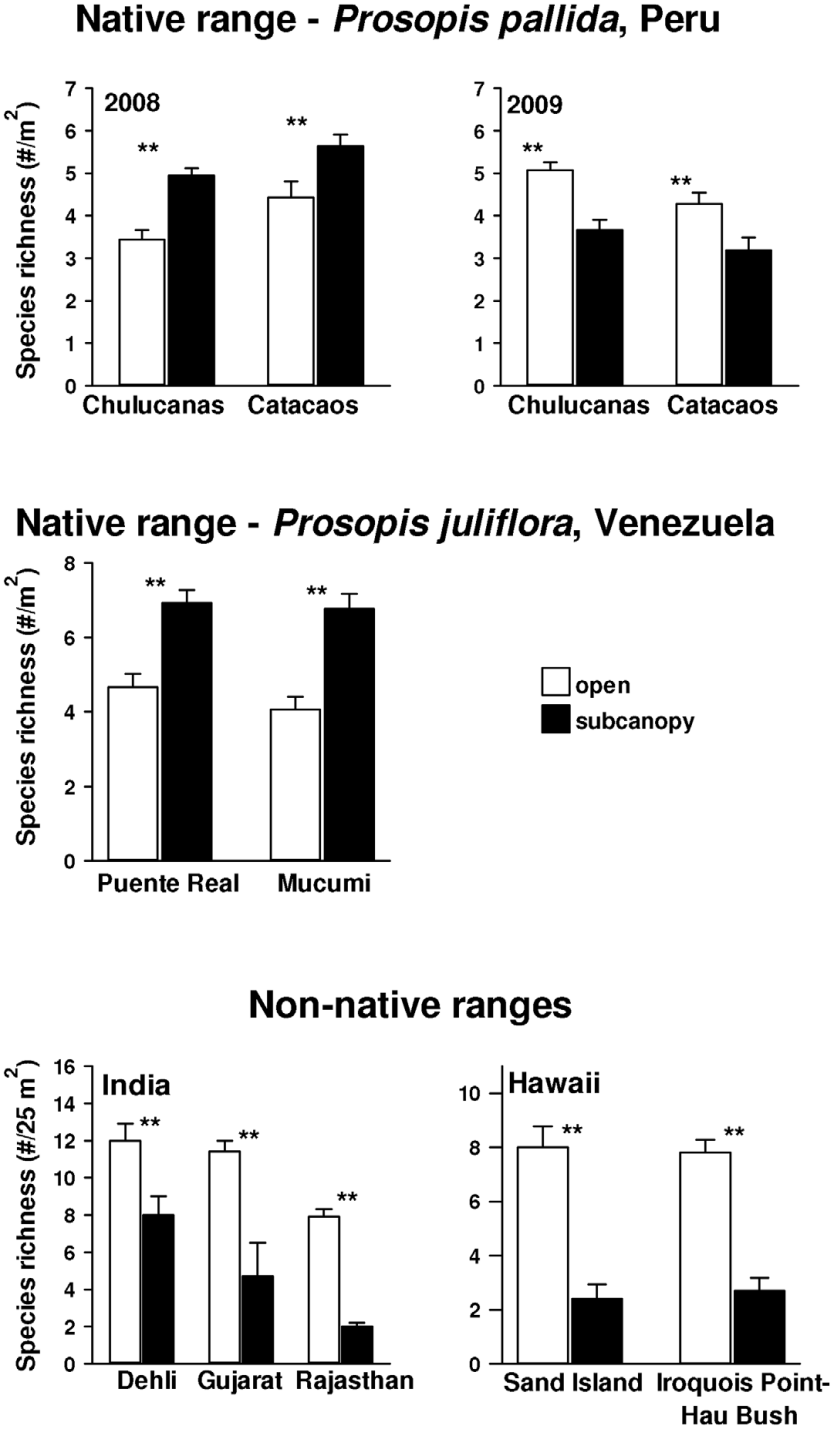
Plant species richness (mean + SE) under canopies of *Prosopis pallida* and nearby open areas in Chulucanas and Catacaos in its native Peru (upper panel); and under canopies of *Prosopis juliflora* and nearby open areas in its native range of Venezuela (middle panel), and non-native ranges of Delhi, Gujarat and Rajasthan in India, and Honolulu in Hawaii, USA two of its non-native ranges (lower panel). Asterisks above paired bars were derived from independent *t* tests except Rajasthan where Mann-Whitney U test (p<0.05) was used.

Plant species richness in the understory of *P. juliflora* in its native range of Venezuela was 48% and 63% higher than in nearby open areas, at Puente Real and Mucumi, respectively (Figure 2, middle panel; F_understory vs. open_ = 324.0; df = 1,56; p = 0.035). Plant species richness in the understory of *P. pallida* in its native range of Peru was 27% and 44% higher than in nearby open areas, at Chulucanas and Catacaos, respectively in 2008 (Figure 2, upper panel; t _Chulucanas_ = 5.13; df = 1,94; p<0.001; t _Catacaos_ =  = 2.67; df = 1,92; p = 0.011). However, in 2009 richness was 28% and 25% lower than in the open at those same sites (Figure 2, upper panel; t _Chulucanas_ = 2.70; df = 1,70; p = 0.009; t _Catacaos_ =  = 4.678, df = 1, 118, p<0.0001). Analyzed over both years *P. pallida* had no effect on understory richness in its native range in Peru (F_canopy_ = 0.79; df = 1,374; P = 0.778).

In the non-native range of India, species richness in *P. juliflora* invaded areas was 32%, 54% and 78% lower at Delhi, Gujarat, and Rajasthan, respectively, compared to areas without *P. juliflora* (Figure 2, lower panel: t_Delhi_ = 2.72; df = 1,18, p = 0.014; t_Gujarat_ = 3.37; df = 1,12; p = 0.006, and for Rajasthan, Mann-Whitney U test, p = 0.012). The mean effect in India over all three sites was a decrease in species richness of 56%. In habitats invaded by *P. juliflora* in India native species accounted for 92%, 67% and 100% of the total number of species present below *P. juliflora* canopies at sites Delhi, Rajasthan and Gujarat, respectively. In Honolulu, Hawaii species richness in *P. juliflora* invaded areas was 70% and 65.3% lower at Sand Island and Iroquis point, respectively (Figure 2, lower panel: t_Sand Island_ = 5.93, df = 1,18, p<0.001; t_Iroquis point_ = 7.49; df = 1,18, p<0.001). In Hawaii, however, more than 90% of understory species were non-native.

### Effects of *Prosopis* Congeners on Species Richness

**Figure 3:**
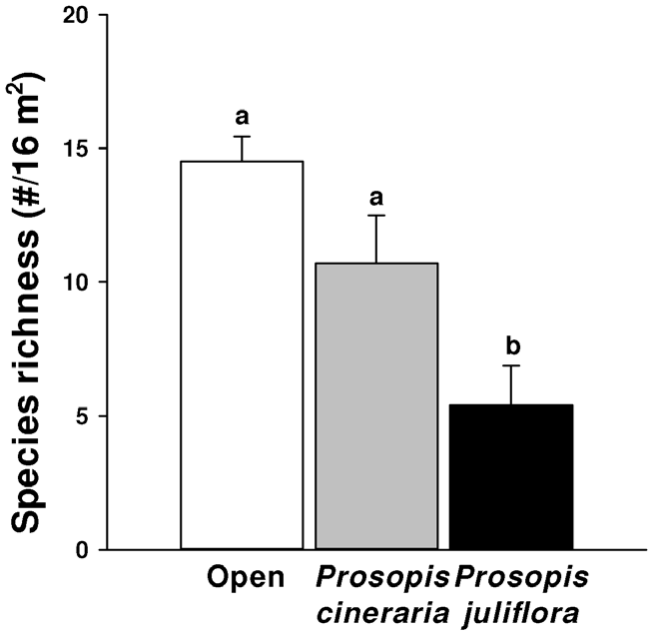
Plant species richness (mean + SE) beneath canopies of *P. juliflora,* its native congener *P. cineraria*, and from open areas. Differences in plant species richness beneath *P. cineraria* and *P. juliflora* canopies and open areas were tested using one way-ANOVA and post-hoc Tukey’s test (p<0.05).

At a fourth site in India, species richness did not differ beneath canopies of *P. cineraria* relative to open areas, but species richness was 63% lower under *P. juliflora* canopies than in the open and 50% lower than under *P. cineraria* canopies (Figure 3; F = 10.018; df = 2,27; P<0.001).

### Effects of Congeners on Soil Chemistry

**Figure 4:**
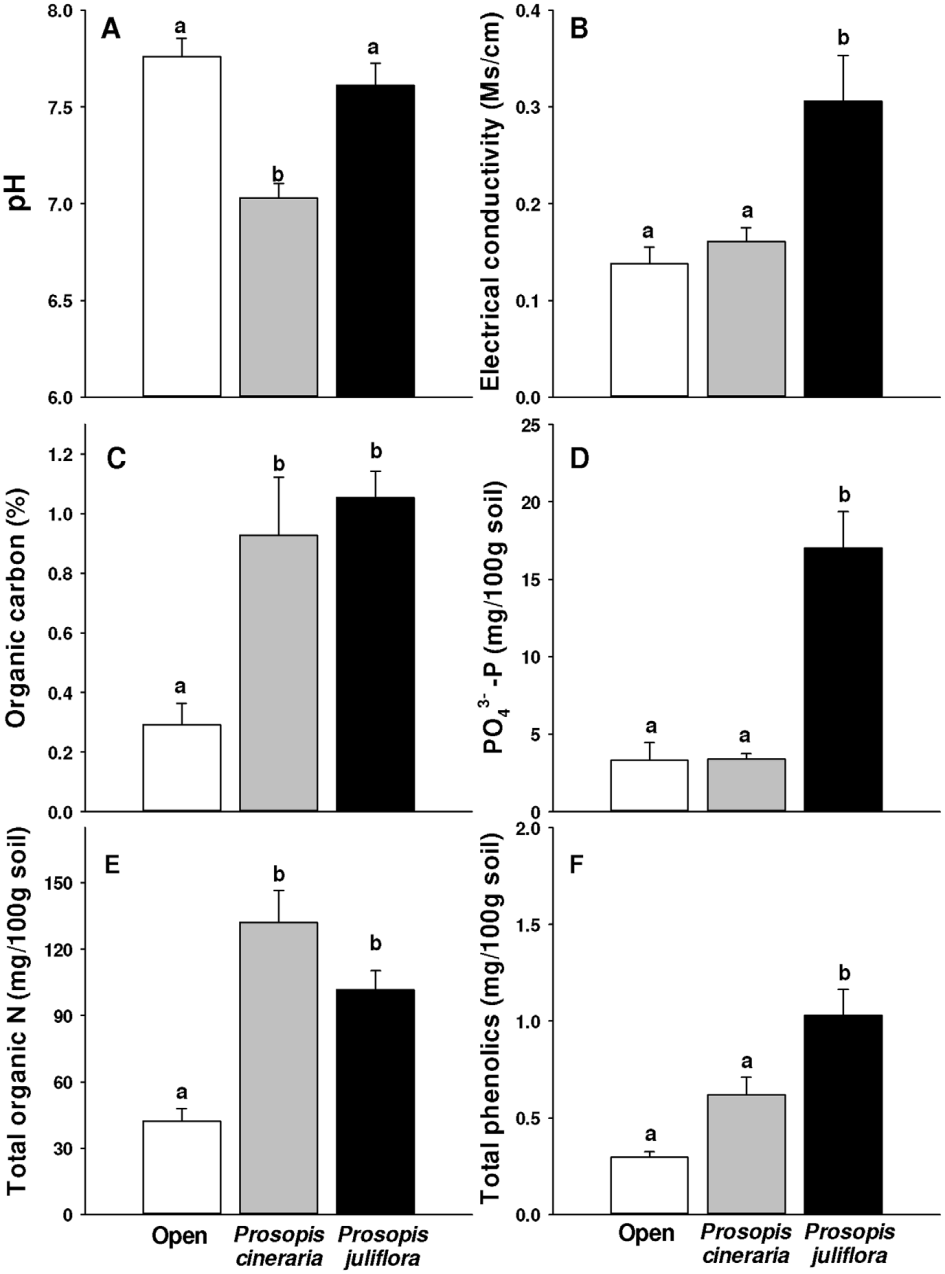
Chemical properties – pH (A), electrical conductivity (B), organic carbon (C), PO_4_^3–^Phosphorus (D), total organic nitrogen (E) and total phenolic content (F) of soil from the rhizosphere of *Prosopis juliflora*, *Prosopis cineraria* and soil from adjacent open area in Punjab, India. Error bars represent +1SE of mean. Different letters above bars indicate significant differences (one way ANOVA, post-ANOVA Tukey’s test; p<0.05).

The pH of *P. cineraria* soil was significantly lower than that of soil in the open (Figure 4A: F_pH_ = 15.914; df = 2,27; p<0.0001, Tukey, p<0.0001) and *P. juliflora* soil (Tukey, p = 0.001) but that of *P. juliflora* soil did not differ from soil in open (Tukey, p = 0.525). As compared to soil in open, organic carbon and nitrogen were significantly higher in *P. juliflora* (Figure 4C; F_Organic carbon_ = 9.798; df = 2,27; p = 0.001, Tukey, p = 0.001; Figure 4E: F_Nitrogen_ = 19.897; df = 2,27; p<0.0001, Tukey, p = 0.001) and *P. cineraria* soils (organic carbon: Tukey, p = 0.005; nitrogen: Tukey’s test, p<0.0001) but did not differ significantly between the two congeners (organic carbon: Tukey, p = 0.775; nitrogen: Tukey, p = 0.109). Electrical conductivity, exchangeable PO_4_^3–^P and total phenolic content were significantly higher in soil from under *P. juliflora* than soil from under *P. cineraria* (Figure 4B: F_Electrical conductivity_ = 9.105; df = 2,27, p = 0.001, Tukey, p = 0.006; Figure 4D: F_Phosphorus_ = 26.574; df = 2,27, p<0.0001, Tukey, p<0.0001: Figure 4F: F_Phenolics_ = 13.711; df = 2,25, p<0.0001, Tukey, p = 0.020) or from the open (electrical conductivity: Tukey, p = 0.001; exchangeable PO_4_^3–^P: Tukey, p<0.0001; total phenolic content: Tukey, p<0.0001). These factors did not differ significantly between *P. cineraria* soil and soil from the open (electrical conductivity: Tukey, p = 0.847; exchangeable PO_4_^3–^P: Tukey, p = 1.000; total phenolic content: Tukey, p = 0.084).

### Effects of Leaf Litter from *Prosopis* Congeners on Other Species

**Figure 5:**
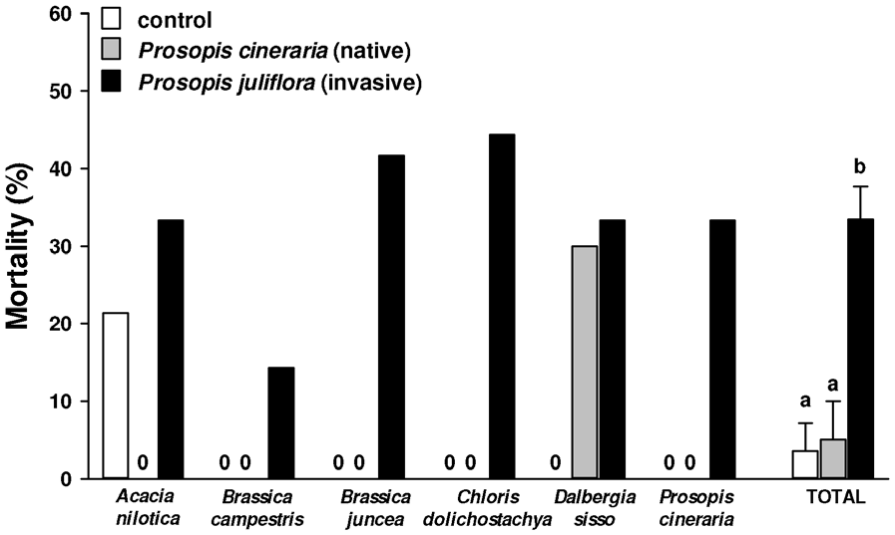
Mortality (mean + SE) of different species - *Acacia nilotica, Brassica campestris, Brassica juncea, Chloris dolichostachya, Dalbergia sissoo* and *Prosopis cineraria* - when grown in soil amended with leaf litter of *Prosopis juliflora* (black box), *P. cineraria* (gray box) or without litter (white box). Different letters above bars indicate significant differences (one way ANOVA, post-ANOVA Tukey’s test; p<0.05).

Leaf litter from the Indian native *P. cineraria* increased the mortality of *Dalbergia sissoo*, but had no effect on any other native Indian species (Figure 5). In contrast, leaf litter from the invasive *P. juliflora* increased the mortality of all six native Indian species from 14–44%. The mean mortality of Indian species when exposed to litter from *P. cineraria* (5±5%) was not significantly different than that in the control (ANOVA, F_treatment_ = 15.09; df = 2,15; P<0.001; Tukey P = 0.970), but much lower than the mean mortality (33±4%) of plants treated with *P. juliflora* litter (Tukey P = 0.001).

### Effects of Leaf Leachate from *Prosopis* Congeners on Other Species

**Figure 6:**
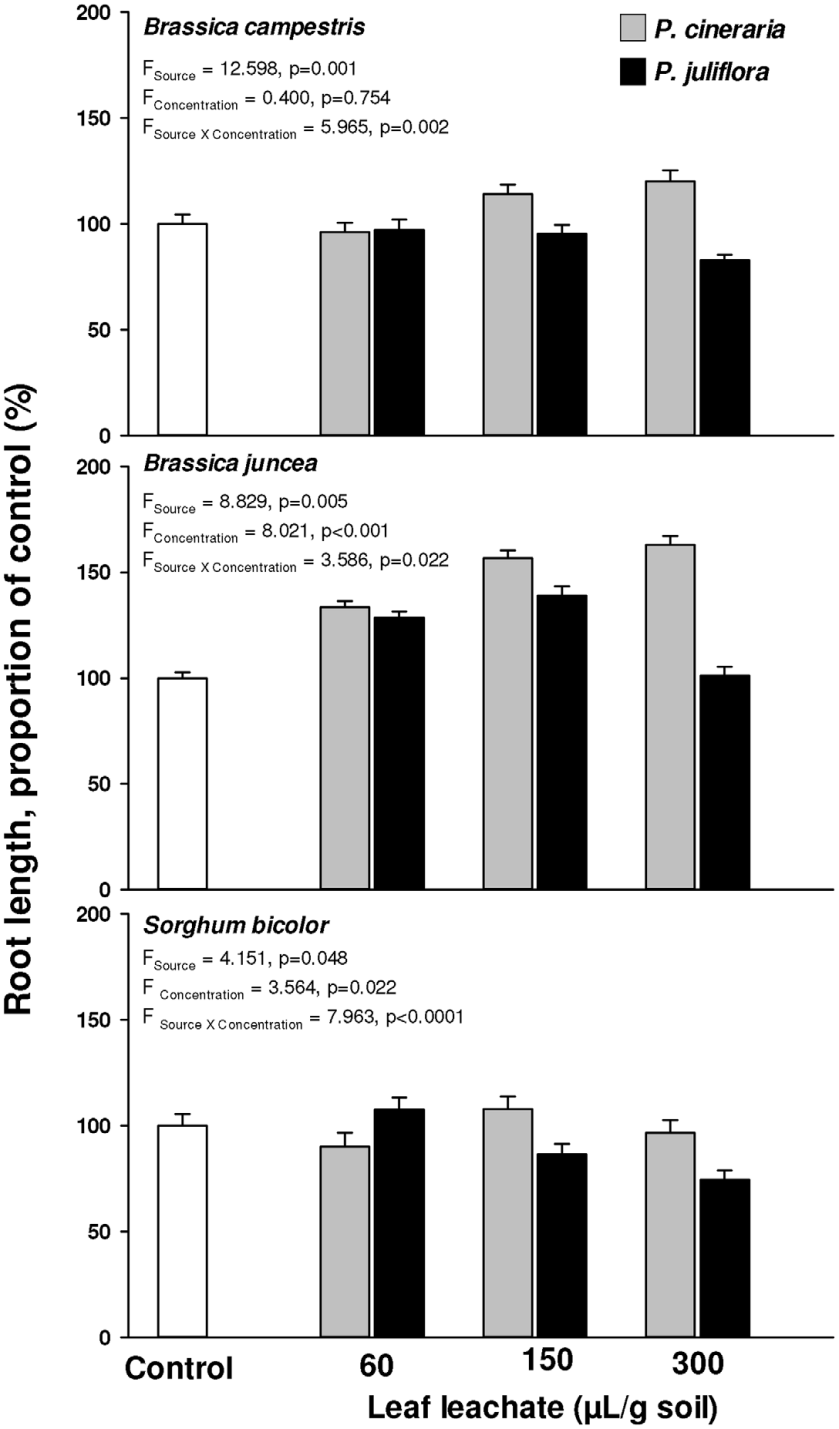
Root length (proportion of control, %) of *Brassica campestris* (upper panel), *B. juncea* (middle panel) and *Sorghum bicolor* (lower panel) seedlings grown in soil treated with different amounts (60, 150 and 300 µL/g soil) of *Prosopis cineraria* (gray bars) or *Prosopis juliflora* (black bars) leaf leachate. Soil treated with distilled water served as untreated control (white bars). Error bars represent +1SE of mean. F and P values are shown for two way ANOVAs.

As compared to control, leachate from the leaves of the native *P. cineraria* significantly enhanced the root growth of *B. campestris* at 150 (Figure 6 upper panel; t_0 vs. 150_ = −2.561; df = 1,10; p = 0.028;) and 300 µL/g soil concentrations (t_0 vs. 300_ = −2.955; df = 1,10; p = 0.014), and of *B. juncea* at 60 (Figure 6 middle panel; t_0 vs. 60_ = −3.506; df = 1,10; p = 0.006), 150 (t_0 vs. 150_ = −4.759, df = 1,6; p = 0.003), and 300 µL/g soil concentrations (t_0 vs. 300_ = −3.067; df = 1,6; p = 0.025). The root length of *S. bicolor* seedlings grown in *P. cineraria* leaf leachate amended soil was not significantly different from control at any tested concentration (Figure 6 lower panel; t_0 vs. 60_ = 1.475; df = 1,10; p = 0.171; t_0 vs. 150_ = −1.370, df = 1,10; p = 0.201; t_0 vs. 300_ = 0.496; df = 1,10; p = 0.631). In contrast, leachate from the leaves of the invasive *P. juliflora* significantly reduced the root growth of *B. campestris* as compared to control at the highest concentration (Figure 6 upper panel, t_0 vs. 300_ = 3.893, df = 1,10, p = 0.003) and of *S. bicolor* at 150 (Figure 6 lower panel, t_0 vs. 150_ = 3.006, df = 1,10, p = 0.013) and 300 µL/g soil concentrations (t_0 vs. 300_ = 6.226, df = 1,10, p<0.0001). *Brassica juncea* root growth increased in soil treated with 60 (Figure 5 middle panel; t_0 vs. 60_ = −2.477, df = 1,10; p = 0.033) and 150 µL/g soil concentrations (t_0 vs. 150_ = −3.693, df = 1,10, p = 0.004) of *P. juliflora* leachate and was not affected at highest concentration (t_0 vs. 300_ = 0.002, df = 1,10, p = 0.999).

Root length of seedlings of all 3 species grown in soil amended with leaf leachate from *P. juliflora* was significantly lower than that of seedlings grown in *P. cineraria* leaf leachate-amended soil at similar concentrations i.e. 150 (Figure 6 upper panel; t*_B. campestris_*_ = _2.764; df = 1,10; p = 0.020; t*_S. bicolor_*_ = _3.116; df = 1,10, p = 0.011) and 300 µL/g soil (t*_B. campestris_*_ = _5.573; df = 1,10; p<0.001; t*_B. juncea_*_ = _2.936; df = 1,10; p = 0.015; t*_S. bicolor_*_ = _4.963; df = 1,10; p = 0.001), with the exception of *B. juncea* at 150 µL/g soil (t*_B. juncea_*_ = _1.151; df = 1,10; p = 0.277). However at 60 µL/g soil concentration, there was no significant difference in root growth of all 3 species grown in *P. juliflora* leaf leachate-amended soil compared to those in *P. cineraria* leaf leachate-amended soil.

There was a significant interaction effect between leachate source and concentration on root growth for all 3 species (Figure 6). We also found significantly negative correlations between *P. juliflora* leachate concentration and root growth of *B. campestris* (r = −0.681, p<0.001) and *S. bicolor* (r = −0.476, p = 0.019), however no significant correlation was observed in case of *B. juncea* (r = −0.060, p = 0.779). In contrast, significant positive correlations between *P. cineraria* leachate concentration and root growth of *B. campestris* (r = 0.594, p = 0.002) and *B. juncea* (r*_ = _*0.589, p = 0.002) were observed, however no significant correlation was observed in case of *S. bicolor* (r = 0.084, p = 0.695).

### Effect of Microbes in Litter of *Prosopis* Congeners on Other Species

Bacteria and fungi isolated from the leaves of *P. julifora* and *P. cineraria*, when applied directly to other species, killed almost all *B. juncea* and *C. dolichostachya* seedlings precluding statistical analysis, but there were no discernible patterns in mortality for isolates from the two congeners. Fungal isolates from *P. juliflora*, applied as a group, killed 97% of *B. juncea* and 98% of *C. dolichostachya* seedlings (data not shown). Bacterial isolates from *P. juliflora*, applied as a group, killed 100% of *B. juncea* and 94% of *C. dolichostachya*. Fungal isolates from *P. cineraria*, applied as a group, killed 98% of *B. juncea* and 100% of *C. dolichostachya* (data not shown). Bacterial isolates from *P. cineraria*, applied as a group, killed 94% of *B. juncea* and 99% of *C. dolichostachya*.

### Chemical Characteristics of Soil Amended with Leaf Leachates of Two Congeners

Both treatments, *P. juliflora* leaf leachate and *P. cineraria* leaf leachate, resulted in a significant decrease in pH and an increase in EC, OC, PO_4_^3–^P, and total organic N of soil with the effect of former treatment being significantly higher than latter (Table S1). *Prosopis cineraria* leaf leachate treated soil did not differ significantly for PO_4_^3–^P from untreated soil (Table S1).

### Effects of Leaf Leachate and Leaf Litter on Soil Phenolic Content

**Figure 7:**
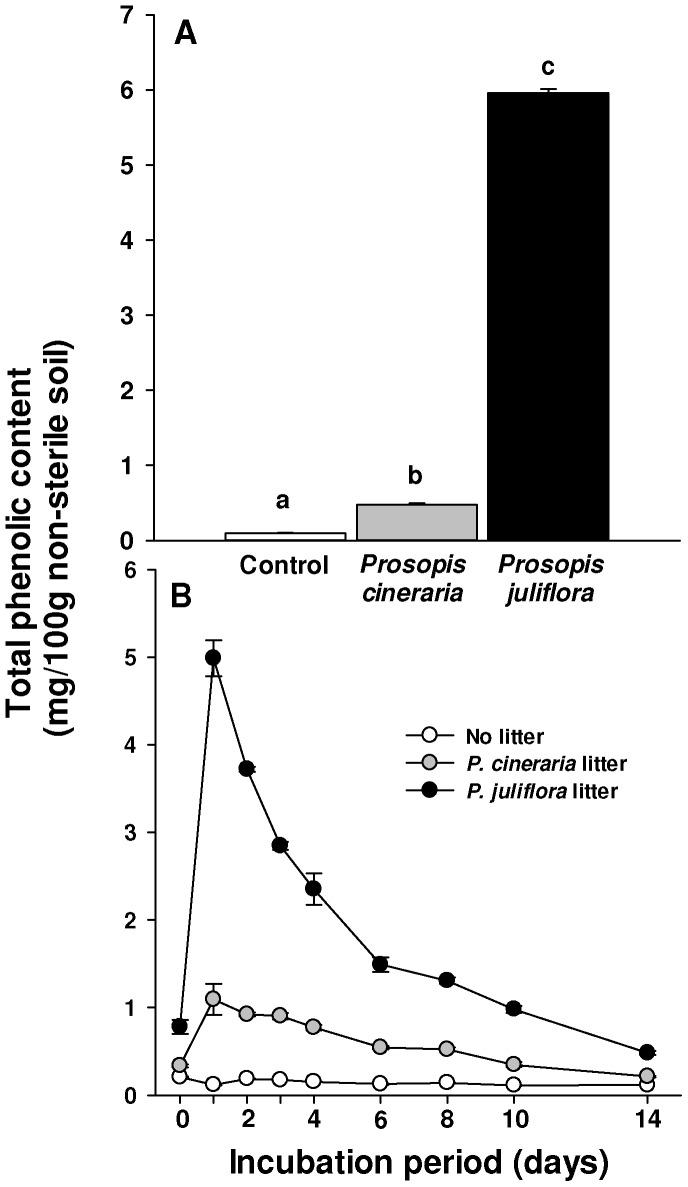
Total phenolic content (mean + SE) of soil amended with *Prosopis cineraria* (gray bar) and *P. juliflora* (black bar) leaf leachates and unamended soil (control, white bar). (A) Different letters above bars indicate significant differences (ANOVA, post-ANOVA Tukey’s test; p<0.05). **(B)** Total phenolic content (mean ± SE) of soil treated with no litter (white circles), *P. cineraria* (gray circles) or *P. juliflora* leaf litter (black circles) at rate of 12 mg/g soil, and incubated at 30–34°C under 12 h/12 h light/dark cycle for 0, 1, 2, 3, 4, 6, 8, 10 and14 days.

Soil amended with leaf leachate of *P. juliflora* had 12.4 times higher values of total phenolics (5.956±0.054 mg/100 g soil) than soil amended with *P. cineraria* (0.479±0.019 mg/100 g soil) and 60.2 times higher concentrations than unamended soil (0.099±0.004 mg/100 g soil; F = 9734.99; df = 2,15; p<0.015; Figure 7A). The total phenolic content of soil amended with leaf litter from *P. juliflora* was significantly higher than soil amended with *P. cineraria* litter and unamended soil, from 0 d to 14 d of incubation (Figure 7B, Table S2).

### L-tryptophan Recovery from Leaf Leachate Amended Soil

L-tryptophan concentrations in the leachates of leaves of *P. juliflora* (4.92±0.05 µg/ml leaf leachate) was 73.1% greater than that of *P. cineraria* (1.32±0.014 µg/ml leaf leachate) (t = 70.509, df = 4, p<0.0001). Over 40% (0.79±0.006 µg/g soil) of the originally added 1.968 µg/g soil of L-tryptophan from *P. juliflora* leachate, was recovered from soil immediately after application, but we could not detect L-tryptophan in soil amended with *P. cineraria* leachates.

## Discussion

*Prosopis juliflora* is among the most invasive species in hot semiarid and arid regions of the world, and our results indicate that this invader has substantially stronger impacts on native diversity in two non-native ranges than in its native range of Venezuela where *P. juliflora* had facilitative effects and in the native range of Peru where *P. pallida* (often misidentified as *P. juliflora* [5]) had overall neutral effects. Despite the widely documented and very strong impacts of invasive species on natives [36], [37], to our knowledge only two other studies have quantified the impact of an invasive species on the productivity or diversity of its neighbors in the field in both its native and non-native ranges. Inderjit et al. [38] found that the canopies of *Ageratina adenophora*, a widespread and aggressive subtropical invader, had facilitative effects on other species in its native Mexico but highly inhibitory effects in its non-native ranges in China and India. These differences were correlated with differences in the allelopathic effects of volatile organic compounds on species native to the different ranges. Callaway et al. [2] found that the biomass of native species in *Acroptilon repens* stands was 25–30 times lower in the non-native range than in the native range. It is important to note that lower plant species richness in plots invaded by *P. juliflora* compared to plots not yet invaded by it, could be due to the negative impact of the invader or colonization of sites that inherently have low species richness; mechanisms that cannot be separated through measurements of correlative patterns. However, consistently stronger negative canopy effects of *P. juliflora* in two non-native ranges compared to its native range, differences in canopy effects between congeners in the non-native range, and direct comparisons of litter from the congeners support the hypothesis that *P. juliflora* is at least in part a “driver” of decreased diversity rather than a “passenger” responding to other factors that also decrease diversity [39]. Strong negative impacts of *P. juliflora* on the richness, evenness and densities of other plant species have also been reported in the United Arab Emirates where it is also invasive [20]. The observed effects of *P. juliflora* canopies on species richness are less likely to be due to higher stand densities because similar negative canopy effects of *P. juliflora* on species richness have also been observed at lower stand densities (unpublished work).

Congeneric comparisons demonstrated stronger impacts of the invasive *P. juliflora* than the native *P. cineraria* on plant diversity. *Prosopis cineraria*, a native Indian species, has been reported to facilitate growth of crop species and is an important species in agroforestry [40]; however, we measured its neutral effects on native diversity. Although biogeographic comparisons have been performed for other invasive plants such as *Ageratina adenophora* [38], *Alliaria petiolata* [41], *Centaurea stoebe* [42], *Acroptilon repens* [2] and *C. diffusa* [43], to our knowledge, this congeneric comparison is unique. Since *P. cineraria* is not an invasive species, we did not study, reciprocally, its positive or negative effects on species exotic to its native range India.

Our results show that like other species of *Prosopis,* both *P. juliflora* and *P. cineraria* form resource islands by accumulating total organic N and organic carbon in their rhizosphere soil. Unlike *P. cineraria*, *P. juliflora* also accumulates soluble salts, exchangeable-P and total phenolics in its soil. However, it appears that the presence of high concentrations of total phenolics in *P. juliflora* soil may override its potential positive effects on soil fertility.

Our study indicates that litter may play at least some role in the impact of *P. juliflora* on soil nutrients, soil phenolics, and the reduction of native species diversity in India. Leaf litter of *P. juliflora* killed far more seedlings of native Indian species than litter of *P. cineraria*. These effects of leaf litter appeared to be through their biochemical effects, results consistent with the Novel Weapons Hypothesis [44]. Bacteria and fungi isolated from *P. juliflora* and *P. cineraria* leaf litter and applied to seedlings killed almost all native Indian species, but there was no hint of a difference in effects between the congeners. Also, much higher amounts of total phenolics in soil amended with *P. juliflora* leaf leachate compared to that amended with *P. cineraria* leachate suggest that the release of more total phenolics into soil may play an important role in the stronger effects of *P. juliflora* litter. In a similar comparison of the phytotoxicity of *P. juliflora* and *P. cineraria* rhizosphere soils, Inderjit et al. [25] reported that *Bambusa arundinacea* seedlings grown in *P. juliflora* soil were smaller than those grown in *P. cineraria* soils, a result that could be due to a higher total phenolic content of *P. juliflora* soils than *P. cineraria* soils, as we measured here. Also, there were much higher amounts of total phenolics leached from *P. juliflora* litter than *P. cineraria* litter over a 14 d period (Figure 7B), suggesting a higher and more continuous supply of phenolics to soil from the litter of *P. juliflora*. Other studies also indicate that *P. juliflora* has substantial allelopathic potential [26], [45], [46]. Allelochemicals could directly affect plant growth or also impact soil microbes [47]. Our unpublished studies suggest that soils amended with *P. juliflora* litter have higher microbial activity compared to soil amended with *P. cineraria* litter. *P. juliflora* allelochemicals might affect plant growth directly and also by influencing microbial activity or microbial communities.

Higher concentrations of allelochemicals in *P. juliflora* compared to *P. cineraria* could be due to higher ploidy levels in *P. juliflora*. Trenchard et al. [48] studied ploidy of 10 *Prosopis* species including *P. juliflora* and found all 9 species to be diploid (2n = 2x = 28) except *P. juliflora* which was tetraploid (2n = 4x = 56). Invasive species are commonly polyploids [49]–[51] and polyploids may have an advantage over their diploid progenitors in having novel phenotypic variation resulting from increased variation in expression of dosage-regulated genes, altered regulatory interactions, rapid genetic and epigenetic changes [52]. The observed higher levels of phenolics or L-tryptophan from the leaf leachate of *P. juliflora* than that of *P. cineraria* could be a novel phenotype resulting from any of these novel gene expression mechanisms following polyploidization.

Although many chemicals or combinations of chemicals could cause the apparent allelopathic effects we measured, we focused on the release of L-tryptophan from litter and in soil because of reports of this chemical in leachates of *P. juliflora* foliage by Nakano et al. [27]. We detected L-tryptophan in leaf leachates of both *P. juliflora* and *P. cineraria*, but the amounts of L-tryptophan in leaf leachate of *P. juliflora* were 73% higher than that of leachate of *P. cineraria*. The presence of higher concentrations of L-tryptophan in soil amended with *P. juliflora* leachate and its rapid disappearance from soil amended with *P. cineraria* leachate supports the potential for L-tryptophan to affect the phytotoxic nature of *P. juliflora* litter. In India the annual leaf litterfall of *P. juliflora* has been reported at 8.1 Mg/ha, and litterfall is highest between February and May [53] when native species (such as *Butea monosperma, Holoptelea integrifolia, Carissa spinarum, Capparis sepiaria, C. decidua, Cenchrus ciliaris* or *Azadirachta indica*) germinate and recruit. Also, the evergreen habit of *P. juliflora* ensures the consistent presence of L-tryptophan in soils. Thus *P. juliflora* litter possesses large amounts of a chemical which is present at far lower amounts in the leaf litter of the native congener *P. cineraria*. There are likely to be other allelopathic or antibiotic chemicals in the litter, leachate or root exudates of *P. juliflora* that we did not measure, but such higher concentrations of a particularly biologically active chemical produced by an invasive species is only partially consistent with the Novel Weapons Hypothesis. The Novel Weapons Hypothesis poses that some successful invaders may possess allelopathic [54], antibiotic [41] or herbivore defense chemicals [55] that are *unique* in the non-native range, giving the invasive species an advantage against evolutionary naive native species [44]. As reported here, small amounts of L-tryptophan also occur in the litter of the Indian native *P. cineraria*. However, the striking differences in the inhibitory effects of litter between the two *Prosopis* species suggest that L-tryptophan is unlikely to be the only active biochemical in the leaves of the invader.

Our results add to a growing body of literature indicating that there is substantial species specificity in the effects of plant-released secondary metabolites [56]. Also, these species-specific interactions suggest that assemblages of plants, or communities, may be less individualistic than often thought (see [57]). Finally, our results suggest that regional evolutionary trajectories exist and that novel competitive mechanisms have the potential to disrupt coevolved interactions among long-associated native species.

## Supporting Information

Table S1 Summary of statistical analysis of pH, electrical conductivity (EC), organic carbon (OC), phosphate-P and total organic N (TON) of soil treated with no leachate (control, C), *P. cineraria* (PC) or *P. juliflora* (PJ) leaf leachate (one-way ANOVA and posthoc Tukey’s test at p<0.05).
(DOCX)

Table S2 Summary of statistical analysis of total phenolic content of soil treated with no litter (control, C), *P. cineraria* (PC) or *P. juliflora* (PJ) leaf litter at rate of 12 mg/g soil, and incubated for 0, 1, 2, 3, 4, 6, 8, 10 and 14 days.
(DOCX)
