# Supplementary material for: Community Impacts of Prosopis juliflora Invasion: Biogeographic and Congeneric Comparisons
Source: PLoS One. 2012 Sep 12;7(9):e44966. doi: 10.1371/journal.pone.0044966 (PMC3440363; doi:10.1371/journal.pone.0044966)
Supplement: Table S1 — Summary of statistical analysis of pH, electrical conductivity (EC), organic carbon (OC), phosphate-P and total organic N (TON) of soil treated with no leachate (control, C), P. cineraria (PC) or P. juliflora (PJ) leaf leachate (one-way ANOVA and posthoc Tukey’s test at p<0.05). (DOCX) [file pone.0044966.s001.docx]

**Table S1. Summary of statistical analysis of pH, electrical conductivity (EC), organic carbon (OC), phosphate-P and total organic N (TON) of soil treated with no leachate (control, C), *P. cineraria* (PC) or *P. juliflora* (PJ) leaf leachate** (one-way ANOVA and posthoc Tukey’s test at p < 0.05).

| Variable | Treatment* | | | ANOVA statistics | | | Tukey's test, p value | | |
| --- | --- | --- | --- | --- | --- | --- | --- | --- | --- |
|  | C | PC | PJ | df | F | p | C vs. PC | C vs. PJ | PC vs. PJ |
| pH | 7.98±0.02^c^ | 7.84±0.01 ^b^ | 7.72±0.00 ^a^ | 2,15 | 97.856 | <0.0001 | <0.0001 | <0.0001 | <0.0001 |
| EC (dS/m) | 0.172±0.002 ^a^ | 0.287±0.002 ^b^ | 0.548±0.003^c^ | 2,15 | 7079.190 | <0.0001 | <0.0001 | <0.0001 | <0.0001 |
| OC (%) | 0.212±0.007 ^a^ | 0.240±0.003 ^b^ | 0.340±0.007 ^c^ | 2,15 | 118.914 | <0.0001 | 0.018 | <0.0001 | <0.0001 |
| P (mg/100g) | 2.68±0.02 ^a^ | 2.76±0.07 ^a^ | 3.34±0.09 ^b^ | 2,15 | 25.881 | <0.0001 | 0.693 | <0.0001 | <0.001 |
| TON (mg/100g) | 40.9±1.0 ^a^ | 45.4±0.3 ^b^ | 54.9±0.6 ^c^ | 2,15 | 111.463 | <0.0001 | <0.001 | <0.0001 | <0.0001 |

*values are mean±se
