# Supplementary material for: Community Impacts of Prosopis juliflora Invasion: Biogeographic and Congeneric Comparisons
Source: PLoS One. 2012 Sep 12;7(9):e44966. doi: 10.1371/journal.pone.0044966 (PMC3440363; doi:10.1371/journal.pone.0044966)
Supplement: Table S2 — Summary of statistical analysis of total phenolic content of soil treated with no litter (control, C), P. cineraria (PC) or P. juliflora (PJ) leaf litter at rate of 12 mg/g soil, and incubated for 0, 1, 2, 3, 4, 6, 8, 10 and 14 days. (DOCX) [file pone.0044966.s002.docx]

**Table S2.** **Summary of statistical analysis of total phenolic content of soil treated with no litter (control, C), *P. cineraria* (PC) or *P. juliflora* (PJ) leaf litter at rate of 12 mg/g soil, and incubated for 0, 1, 2, 3, 4, 6, 8, 10 and14 days.**

| Day | Total phenolics (mg/100g soil)* | | | ANOVA statistics | | | Tukey's test;p value | | |
| --- | --- | --- | --- | --- | --- | --- | --- | --- | --- |
|  | No litter | PC litter | PJ litter | F value | df | p | C vs. PC | C vs. PJ | PC vs. PJ |
| 0 | 0.208±0.005**^a^** | 0.338±0.017**^a^** | 0.780±0.081**^b^** | 39.037 | 2, 9 | <0.001 | 0.192 | <0.001 | <0.001 |
| 1 | 0.122±0.010**^a^** | 1.093±0.177**^b^** | 4.988±0.021**^c^** | 267.319 | 2, 9 | <0.001 | 0.005 | <0.001 | <0.001 |
| 2 | 0.188±0.005**^a^** | 0.923±0.025**^b^** | 3.722±0.029**^c^** | 6987.644 | 2, 9 | <0.001 | <0.001 | <0.001 | <0.001 |
| 3 | 0.174±0.003**^a^** | 0.904±0.037**^b^** | 2.847±0.047**^c^** | 1592.79 | 2, 9 | <0.001 | <0.001 | <0.001 | <0.001 |
| 4 | 0.155±0.005**^a^** | 0.774±0.030**^b^** | 2.351±0.179**^c^** | 116.579 | 2, 9 | <0.001 | 0.006 | <0.001 | <0.001 |
| 6 | 0.128±0.008**^a^** | 0.545±0.020**^b^** | 1.493±0.082**^c^** | 203.298 | 2, 9 | <0.001 | 0.001 | <0.001 | <0.001 |
| 8 | 0.143±0.003**^a^** | 0.522±0.023**^b^** | 1.307±0.036**^c^** | 574.869 | 2, 9 | <0.001 | <0.001 | <0.001 | <0.001 |
| 10 | 0.113±0.005**^a^** | 0.350±0.028**^b^** | 0.981±0.041**^c^** | 241.623 | 2, 9 | <0.001 | 0.001 | <0.001 | <0.001 |
| 14 | 0.115±0.000**^a^** | 0.214±0.010**^b^** | 0.482±0.022**^c^** | 181.698 | 2, 9 | <0.001 | 0.002 | <0.001 | <0.001 |

*values are mean±SE
